# Improving radiologic communication in oncology: a single-centre experience with structured reporting for cancer patients

**DOI:** 10.1186/s13244-020-00907-1

**Published:** 2020-09-29

**Authors:** Tim Frederik Weber, Manuela Spurny, Felix Christian Hasse, Oliver Sedlaczek, Georg Martin Haag, Christoph Springfeld, Theresa Mokry, Dirk Jäger, Hans-Ulrich Kauczor, Anne Katrin Berger

**Affiliations:** 1 Department of Diagnostic and Interventional Radiology, Heidelberg University Hospital, Im Neuenheimer Feld 410, 69121 Heidelberg, Germany; 2 Department of Medical Oncology, National Center for Tumor Diseases (NCT), Heidelberg University Hospital, Heidelberg, Germany

**Keywords:** Neoplasms, Diagnostic imaging, Image interpretation (computer-assisted), Medical oncology

## Abstract

**Objectives:**

Our aim was to develop a structured reporting concept (structured oncology report, SOR) for general follow-up assessment of cancer patients in clinical routine. Furthermore, we analysed the report quality of SOR compared to conventional reports (CR) as assessed by referring oncologists.

**Methods:**

SOR was designed to provide standardised layout, tabulated tumour burden documentation and standardised conclusion using uniform terminology. A software application for reporting was programmed to ensure consistency of layout and vocabulary and to facilitate utilisation of SOR. Report quality was analysed for 25 SOR and 25 CR retrospectively by 6 medical oncologists using a 7-point scale (score 1 representing the best score) for 6 questionnaire items addressing different elements of report quality and overall satisfaction. A score of ≤ 3 was defined as a positive rating.

**Results:**

In the first year after full implementation, 7471 imaging examinations were reported using SOR. The proportion of SOR in relation to all oncology reports increased from 49 to 95% within a few months. Report quality scores were better for SOR for each questionnaire item (*p* < 0.001 each). Averaged over all questionnaire item scores were 1.98 ± 1.22 for SOR and 3.05 ± 1.93 for CR (*p* < 0.001). The overall satisfaction score was 2.15 ± 1.32 for SOR and 3.39 ± 2.08 for CR (*p* < 0.001). The proportion of positive ratings was higher for SOR (89% versus 67%; *p* < 0.001).

**Conclusions:**

Department-wide structured reporting for follow-up imaging performed for assessment of anticancer treatment efficacy is feasible using a dedicated software application. Satisfaction of referring oncologist with report quality is superior for structured reports.

## Key points

- Supported by dedicated software, high-volume utilisation of profoundly structured radiology reports is feasible for general follow-up imaging in cancer patients.
- Report quality is rated better for structured reports than for conventional reports by oncologists.

## Introduction

For patients with solid cancers, the results of imaging examinations are of crucial importance for primary diagnosis and treatment guidance during the further course of disease. Imaging findings do heavily impact on therapeutic decisions and treatment strategies of referring clinicians both in the curative and the advanced tumour situation. For communication of results, written radiology reports are commonly used. Traditionally, free-form narrative reports have been generally used in the radiologic community [1]. Due to the risk of incompleteness and lack of comprehensibility of relevant information, the need for improved and structured reporting has been claimed not only during the last decade [2].

For primary diagnosis and local staging of several tumour entities, disease-specific reporting templates have been proposed by medical societies, e.g. for rectal cancer [1, 3] or pancreatic cancer [1, 4]. Regarding content and presence of key descriptors, superiority of structured reports over conventional reports has been demonstrated for both of these diseases [5–8] as well as for several other malignancies such as prostate cancer [9] and hepatocellular carcinoma [10, 11]. Thus, the advantages of structured reporting for primary diagnosis and initial local staging are well recognised.

However, the vast majority of workload in radiology departments associated with comprehensive cancer centres consists of follow-up imaging of cancer patients with advanced disease to determine efficacy of cancer treatment. For clinical trials, the Response Criteria In Solid Tumours (RECIST) were introduced initially in 2000 for standardisation of response assessment [12]. For clinical routine assessment of tumour patients, there is no common proposal to harmonise layout, content and terminology using structured reporting to date.

We here report the conceptual design, clinical implementation and practical utilisation of a structured oncology report (SOR) dedicated to follow-up of patients with metastatic cancer at a radiology department of a high-volume university hospital. Furthermore, we present the results of an analysis regarding the reporting quality of SOR compared to conventional reports (CR) as assessed by referring oncologists.

## Materials and methods

### Concept of structured oncology reporting

**Fig. 1:**
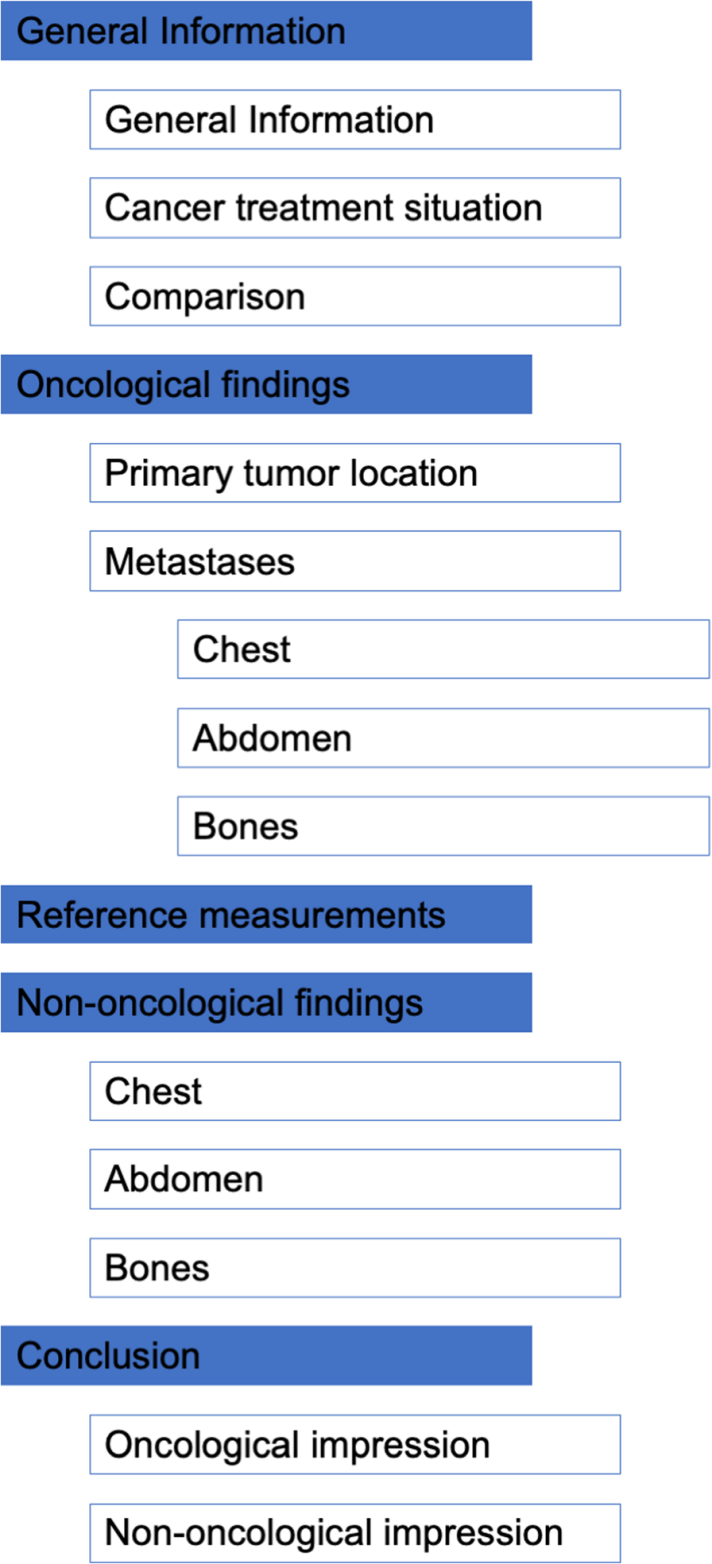
Schematic illustration of layout of structured oncology reports

The concept of structured oncology reporting was interdisciplinarily designed by an expert panel of radiologists (T.F.W., O.S., T.M., H.U.K.) and oncologists (G.M.H., C.S., D.J., A.K.B.) to provide a specific framework to be used in imaging examinations for follow-up of cancer patients with solid tumours. Conceptualisation was based on personal experience and considered available evidence concerning report content preferences [13, 14] and guidelines for tumour response assessment [15]. Aside from standardised content, the concept includes in-house programming and utilisation of a browser-based software application for generating SOR. Figure 1 shows a schematic illustration of the layout of a SOR. SOR is pillared on three main principles that address important criteria of report quality:

#### Standardised layout

The SOR has a consistent organisation and is separated into specific sections. After a section dedicated to assessment of general information concerning imaging and clinical data, the descriptive part of the report contains separate sections for oncological and non-oncological findings, respectively. The content of the section for oncological findings is divided further into subsections for imaging findings regarding the location of the primary tumour and the presence of metastases at different anatomical sites. The conclusion of the report is divided into subsections for oncological impression and non-oncological impression. The oncological impression provides standardised content using uniform terminology (see below).

#### Tabulated documentation of tumour burden

In the SOR, tumour measurements are documented in tables. Reference lesions are selected and measured following the rules provided by RECIST 1.1 [15]. The sum of the diameters of the reference lesions serves as the primary quantitative measure used for response assessment.

#### Standardised conclusion using uniform terminology

The content of the oncological conclusion is standardised concerning structure and vocabulary used for tumour response categorisation.

- Clinical tumour response assessment using a uniform terminology for response categories in due consideration of short-term and long-term imaging (Table 1). Assignment of clinical response categories is thought to consider quantitative measures of tumour burden following rules similar to RECIST 1.1 as well as subjective impressions of tumour burden development. The clinical response category is supplemented by a free-text summary of relevant oncological findings.
- Formal tumour response assessment strictly adhering to RECIST 1.1 in due consideration of baseline and nadir imaging if applicable

**Table 1:** Uniform terminology for tumour response categories

SOR category	SOR threshold criteria	RECIST 1.1 equivalent	RECIST 1.1 threshold criteria*
Significant decrease of tumour burden	≥ 30% decrease in tumour burden and/or qualitative evidence of improvement	Partial response (PR)	≥ 30% decrease in tumour burden (compared with baseline)
Slight decrease of tumour burden	≥ 10–30% decrease in tumour burden and/or qualitative evidence of moderate improvement	n.a.	n.a.
No significant change of tumour burden	Absence of quantitative or qualitative change of tumour burden	Stable disease (SD)	Criteria for PR and PD are not met
Slight increase of tumour burden	≥ 10–20% increase in tumour burden and/or qualitative evidence of moderate worsening	n.a.	n.a.
Significant increase of tumour burden	≥ 20% increase in tumour burden and/or qualitative evidence of remarkable worsening	Progressive disease (PD)	≥ 20% increase in tumour burden (compared with nadir)

*SOR* structured oncology report, *RECIST* response evaluation criteria in solid tumours, *n.a.* not available

*In extracts; for full reference see [13]

### Software application for structured reporting

A software application was programmed to ensure consistency of layout and vocabulary of SOR and to facilitate utilisation in clinical practice. The web browser-based application provides report templates using HyperText Markup Language (HTML) forms. HTML form elements are processed with JavaScript to generate the final report text, which is copied and pasted into the radiology information system (RIS) after completion. Different types of input elements, such as text fields, checkboxes and drop-down lists, are used to ensure uniform terminology on the one hand and to provide space for narrative description of findings on the other. The application can be accessed for review using the following internet link: http://www.targetedreporting.com/sor/.

The template for SOR is designed in such a way that the abovementioned principles of the reporting concept are supported and adherence to reporting formalities is facilitated.

#### Standardised layout

The HTML form sections handling the descriptive parts of oncological and non-oncological findings are set up to largely meet widespread anatomy-based reporting habits, in which the report is traditionally generated from head to toe. That is, for a given anatomic region, oncological and non-oncological findings are entered in the same form block. With entering data into specific input elements, the content is processed and rearranged to occur at the correct position in the final report text output. Pre-formulated phrases are provided as selectable input options to accelerate reporting of additional findings.

#### Tabulated documentation of tumour burden

Input form elements for integers are used for entering reference lesion diameters. The sum of the diameters of the reference lesions and the absolute and relative change of the sum of the diameters in comparison to the prior imaging examination are calculated automatically.

#### Standardised conclusion

The oncological conclusion provides means to ensure that formalities of structure and content including the uniform response terminology are complied with.

### Implementation of standardised oncology reports

The different main elements of SOR were implemented gradually into clinical routine. First, tabulated documentation of reference lesions was integrated into CR. Second, the uniform terminology for response assessment was added. Third, the standardised layout and the whole concept of SOR were introduced into clinical practice with providing the software application. Starting with select key users, utilisation of SOR was increased successively over a time period of three months (training period). After the training period, the whole imaging department was instructed to use SOR for reporting of computed tomography (CT) and magnetic resonance imaging (MRI) examinations performed for follow-up in cancer patients (implementation period). An internal white paper was issued to serve as reporting guideline and reference in daily practice.

For assessment of SOR implementation into clinical routine numbers on utilisation of SOR during the training period and the first year of the implementation period were extracted from the radiology information system.

### Analysis of reporting quality

#### Study design

Reporting quality as assessed by medical oncologists was analysed in a retrospective study design comparing SOR with CR.

#### Report selection for assessing reporting quality

Metastatic colorectal cancer (mCRC) was decided to serve as a pars pro toto of advanced solid tumour diseases. The decision for mCRC was made because mCRC represents one of the most frequent tumour diseases in both men and women and shows a rather uniform pattern of tumour spread facilitating interindividual comparison. Twenty-five CR and 25 SOR of CT scans of chest, abdomen and pelvis performed for follow-up of mCRC patients were extracted from the radiology information system. The 25 CR were obtained from 25 consecutive mCRC patients that were examined prior to implementation of SOR at our institution. The 25 SOR were obtained from another 25 mCRC patients that were examined after implementation of SOR. The number of reports had exploratory character, because data allowing proper sample size calculation on the basis of a comparable type of structured reporting were not available.

#### Layout of conventional reports

CR created prior to introduction of SOR had a non-harmonised format but typically included a section for anatomy-based description of findings and a section for the conclusion. Presentation of tumour measurements was done at the discretion of the radiologist either in-line within the text or using a table format.

#### Rating of reports

CR and SOR of all patients were rated individually by six randomly assigned independent physicians (observers) from the internal department of medical oncology. These included three assistant physicians in medical oncology (with 1 year, 3 years and 4 years of professional experience) and three board-approved medical oncologists (with 12 years, 13 years and 15 years of professional experience). The de-identified reports were presented to the observers in a randomised manner.

Reports were rated using a questionnaire that contained six items addressing different elements of reporting quality (Table 2). Items 1 and 2 addressed the clarity of the presentation of oncological and non-oncological findings. Items 3 and 4 addressed the clarity of the presentation of tumour measurements and tumour response. Item 5 addressed the completeness of the report regarding answering the medical question. Item 6 addressed the overall satisfaction of the observer with the report.

**Table 2:** Questionnaire items addressing reporting quality

1	Oncological findings are presented clearly.
2	Non-oncological findings are presented clearly.
3	Tumour measurements are presented accurately.
4	Tumour response assessment is performed decidedly.
5	The report answers the medical question sufficiently.
6	I am satisfied with the radiology report.

For rating of the questionnaire items, a 7-point scale was used with a score of 1 representing the best score. A score lower than or equal to 3 was defined as a positive rating.

#### Ethics approval

The analysis of reporting quality was approved by the ethics committee of the University of Heidelberg with a waiver of informed consent for patients whose reports were used (S-082/2018). The observers selected for rating the reports consented in written form to study participation and use of their personal data.

#### Statistics

Scores were compared between SOR and CR using the Mann-Whitney *U* test. For assessment of differences concerning the proportion of positive ratings (score lower than or equal to 3 compared with score greater than 3) between board-approved medical oncologists and residents as well as between SOR and CR the chi-squared test was used. Interrater agreement was determined using the agreement coefficient 2 (AC2) according to Gwet [16]. In contrast to Cohen’s Kappa, Gwet’s coefficients have been specifically developed for analysis of agreement between more than two raters and are less vulnerable to the interrater agreement paradoxes described by Cicchetti and Feinstein [17]. Levels of agreement were defined using the classification of Landis and Koch [18]. A *p* value below 0.05 was considered to indicate statistical significance. Statistical calculations were performed using Excel for Mac version 16 (Microsoft Corporation, Redmond, USA) and SPSS version 25 (IBM, Armonk, USA).

## Results

### Implementation of SOR into clinical practice

**Fig. 2:**
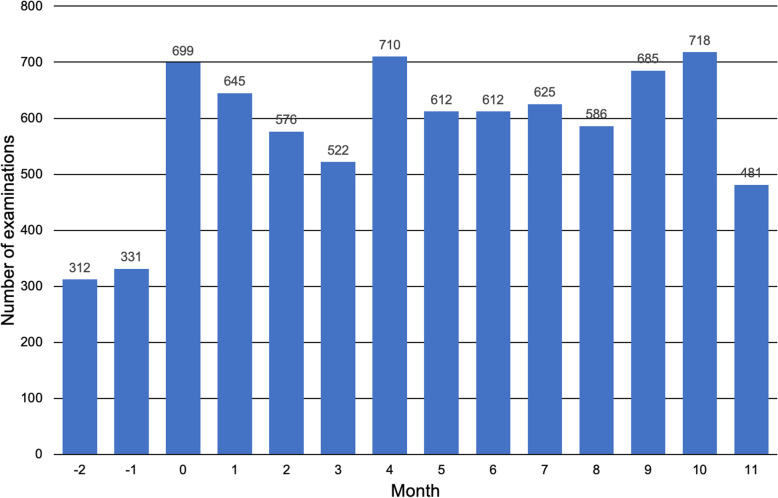
Numbers of imaging examinations reported using structured oncology reports. Month 0 indicates time point of department-wide roll out of structured oncology reports. SOR, structured oncology report

**Fig. 3:**
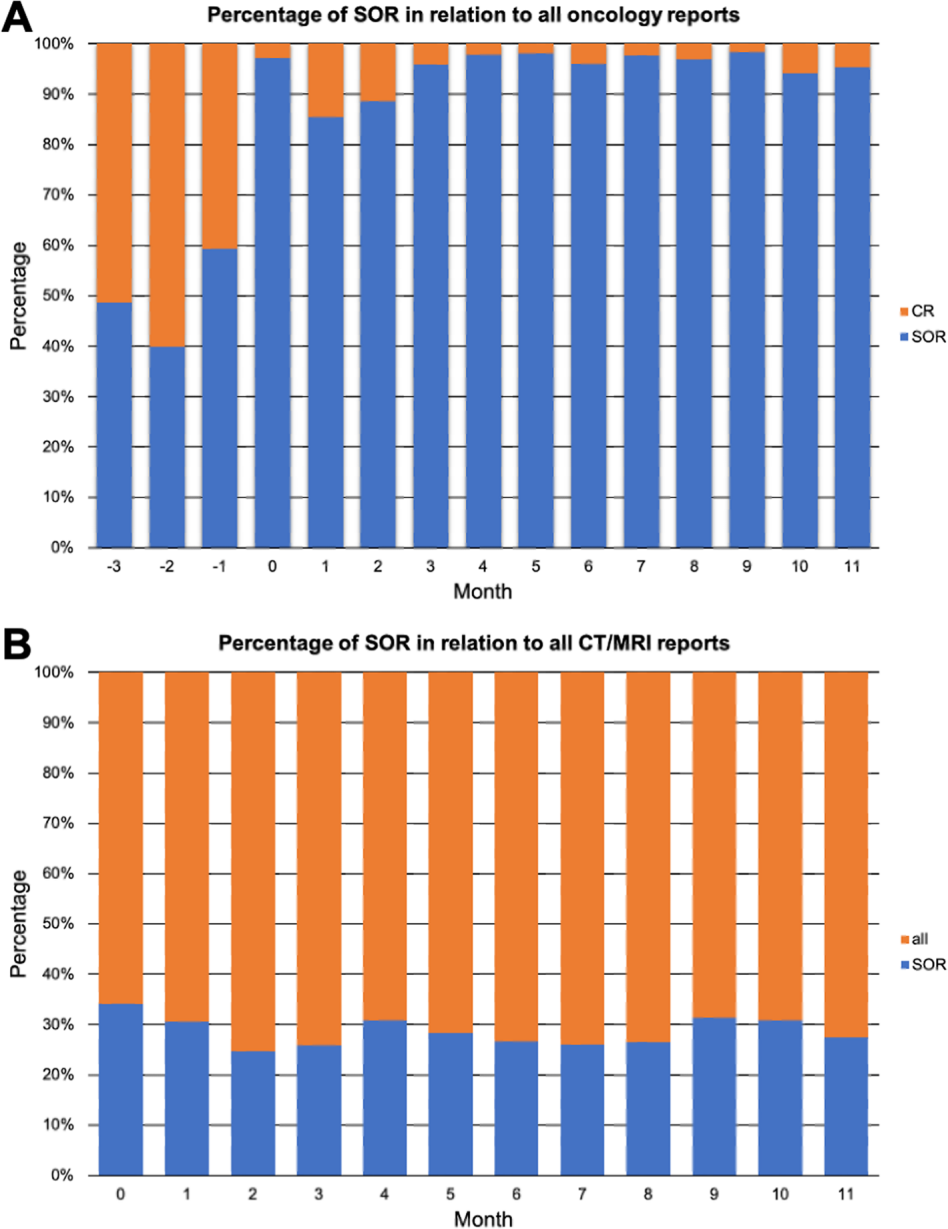
Percentage of structured oncology reports used for oncological follow-up imaging. **a** The proportion of SOR to all reports generated for a representative division of the department for medical oncology. **b** The proportion of SOR to all reports generated for scans at the CT and MRI scanners used for body imaging. Month 0 indicates time point of department-wide roll out of structured oncology reports. SOR, structured oncology report; CR, conventional report

In total, 7471 imaging examinations were reported with SOR after department-wide roll out of SOR in the first 12 months of the implementation period. Figure 2 shows the absolute number of imaging examinations that were reported with SOR on a per-month basis for the training period and the first 12 months of the implementation period. The proportion of SOR in relation to all reports created for one division of the department of medical oncology increased from 49% for the training period to 95% for the first 12 months of the implementation period (Fig. 3a). Of note, these numbers also include examinations of cancer patients that were not performed in the context of oncological follow-up. The proportion of SOR in relation to all reports (oncological and non-oncological reports) created for examinations at CT and MRI scanners used for body imaging was 29% during the first 12 months of the implementation period (Fig. 3b). Multi-region examinations such as CT of chest, abdomen and pelvis are counted as one examination.

### Analysis of reporting quality

The scores for items 1 and 2 were significantly better for SOR regarding both oncological findings (item 1; 1.96 ± 1.16 versus 3.02 ± 1.82; *p* < 0.001) and non-oncological findings (item 2; 2.33 ± 1.41 versus 2.88 ± 1.75; *p* = 0.018). The proportion of positive ratings (score ≤ 3) was higher for SOR concerning clarity of oncological findings (90% versus 69%; *p* < 0.001) as well as clarity of non-oncological findings (85% versus 68%; *p* < 0.001).

The scores for items 3 and 4 were significantly better for SOR regarding both presentation of tumour measurements (item 3; 2.03 ± 1.22 versus 3.39 ± 2.05; *p* < 0.001) and definition of tumour response categories (item 4; 1.73 ± 1.03 versus 2.89 ± 2.00; *p* < 0.001). The proportion of positive ratings (score ≤ 3) was higher for SOR concerning presentation of tumour measurements (89% versus 61%; *p* < 0.001) as well as definition of tumour response categories (92% versus 69%; *p* < 0.001).

The score for item 5 was significantly better for SOR regarding the sufficiency of the reports for answering the medical question (1.70 ± 1.02 versus 2.74 ± 1.82; *p* < 0.001). The proportion of positive ratings (score ≤ 3) was higher for SOR (94% versus 72%; *p* < 0.001).

Overall satisfaction scores were significantly better for SOR (item 6; 2.15 ± 1.32 versus 3.39 ± 2.08; *p* < 0.001). The proportion of positive ratings (score ≤ 3) was higher for SOR (86% versus 63%).

Averaged over all questionnaire item scores were significantly better for SOR (1.98 ± 1.22 versus 3.05 ± 1.93; *p* < 0.001). The proportion of positive ratings (score ≤ 3) was higher for SOR (89% versus 67%; *p* < 0.001). The proportion of items that were rated positively by all six readers was higher for SOR (53% versus 9%; *p* < 0.001). Interrater agreement was excellent for SOR (AC2 = 0.812) and moderate for CR (AC2 = 0.561).

The proportion of positive ratings of SOR tended to be higher for board-approved medical oncologists than for assistant physicians (91% versus 87%; *p* = 0.05). The proportion of positive ratings of conventional reports was higher for assistant physicians than for board-approved medical oncologists (76% versus 58%; *p* < 0.001).

**Fig. 4:**
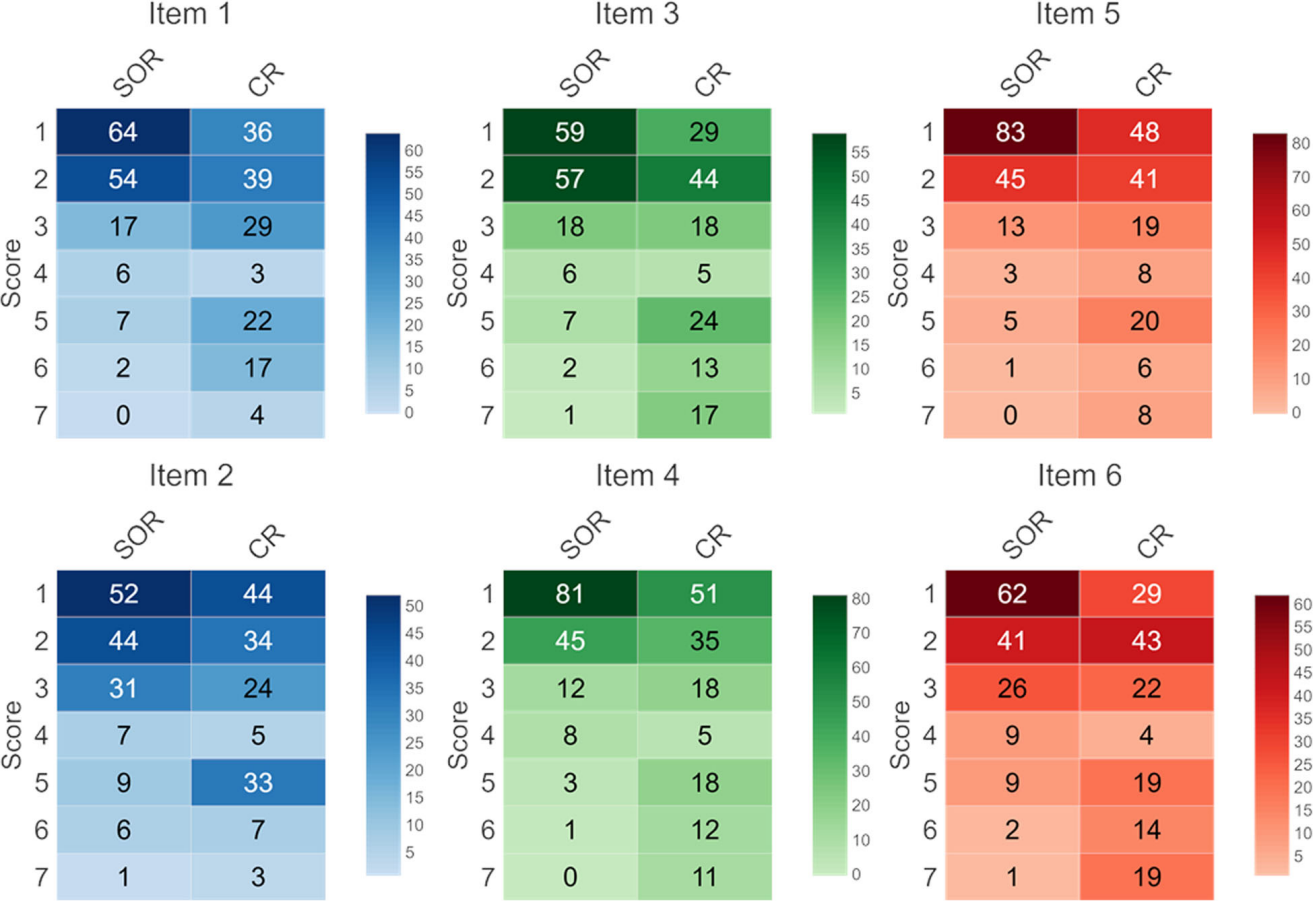
Heatmaps of score distribution for questionnaire items. Numbers in boxes indicate numbers of ratings per score considering all observers. SOR, structured oncology reports; CR, conventional reports

**Fig. 5:**
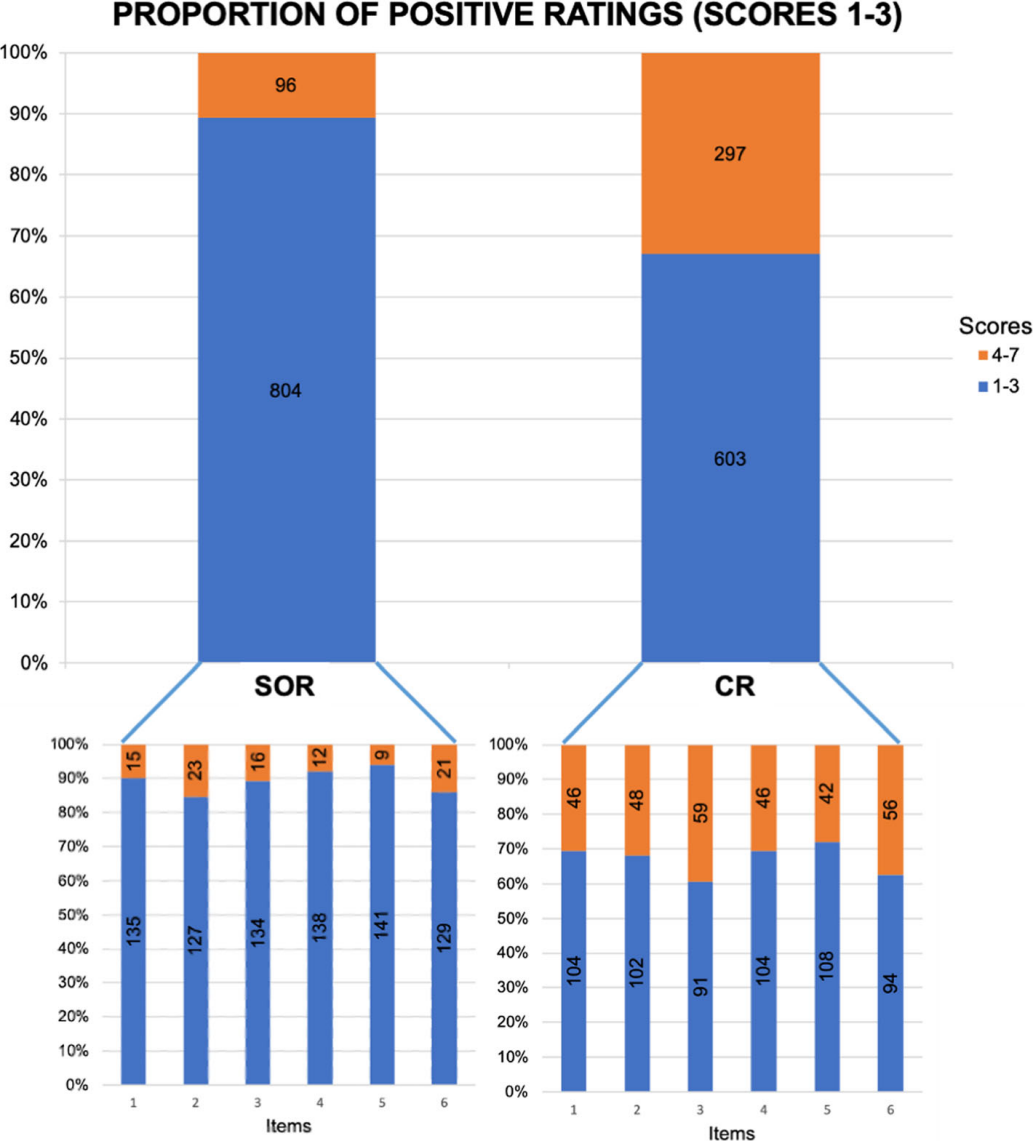
Proportions of positive ratings. SOR, structured reports; CR, conventional reports

Figure 4 shows heat maps of the distribution of scores assigned to each item. Figure 5 illustrates the proportions of positive ratings for all items including all readers.

## Discussion

We have demonstrated the feasibility of a department-wide implementation of an elaborated structured reporting concept for clinical response assessment in patients with advanced solid tumour diseases. SOR was designed primarily to be used for oncological follow-up of metastatic disease and aims at providing a comprehensive diagnostic means to support the oncologist in making the most appropriate treatment decision in due consideration of imaging and clinical course. As the available reporting items resemble the structure of the TNM classification, SOR may also be used for initial staging of newly diagnosed cancers. However, tumour-specific reporting templates are considered more appropriate for initial staging of most cancers in order to reliably address the relevant questions. Within a few months after introduction into clinical practice, SOR has replaced CR and now represents the backbone of oncological imaging in our high-volume cancer centre. Establishing SOR goes hand in hand with introduction of an information technology (IT) solution programmed in-house for report creation using web browser forms. Analysis of referring physicians’ satisfaction shows superiority of SOR over CR regarding different elements of report quality. This applies to both senior medical oncologists and assistant physicians in training.

The European Society of Radiology has published several practice guidelines addressing quality standards of radiology reports [19–22]. However, there is ongoing discussion on how to define structured reporting [23]. With reference to Weiss and Bolos [24], the European Society of Radiology identifies three levels of complexity of structured reporting [22, 24]: The first level comprises a structured format with headings and subheadings. The second level is a consistent organisation ensuring that all relevant aspects of an imaging study are considered. The third level uses a standardised language to improve communication and reusability of radiology reports. Reasons for using structured reporting include improvement of reporting quality as well as datafication and accessibility of radiology reports for scientific purposes [22]. A distinction between standardised reporting and structured reporting has been suggested [23]: Standardised reporting is proposed to represent a means of streamlining the medical content of a radiological report. Structured reporting is proposed to include particular IT to arrange the radiology report [23]. An IT-based reporting tool is thought necessary to support the reporting radiologist by ordering the report into a certain layout (level 1) and by providing predefined medical content (level 2) [23]. Considering this, our reporting concept is supposed to meet the criteria of top-level structured reporting as a specific computer application is being used to provide report templates, to arrange the content and to assist in using standardised language for clinical response assessment. Aside from a uniform terminology in the conclusion, description of findings in the sections for oncological and non-oncological observations is based deliberately on narrative free-text in order to provide the reporting radiologist with sufficient degrees of freedom to portray the individual abnormalities. Available data suggest that the combination of free-text and predefined phrasing options is superior to conventional reporting and beneficial for interdisciplinary communication [25]. Bearing this in mind, specification of an objective tumour response category in the conclusion is complemented by a summarising description of oncological findings that is used to also convey a subjective impression of tumour load and gives room to indicate recommendations for further clinical decision-making.

A survey on expectations regarding the radiology report as seen by radiologists and referring clinicians showed need for improving reporting habits [19]. Referring physicians generally prefer structured reporting with an itemised layout over conventional reports [13], although studies with contrary results are available as well [26]. Concerning oncological imaging, most oncologists feel that conventional reports are not sufficient for assessing tumour burden in oncological patients [27] and presentation of tumour measurement data in a tabulated form is preferred [14]. For decision-making in patients with malignant lymphoma, structured reports have proven superior to conventional reports [28]. In line with these findings, our study shows that presentation of tumour measurements, definition of tumour response and satisfaction with answering the medical question were rated better for structured reports than for conventional reports.

In terms of practical implementation, the successful integration of department-wide structured reporting programmes using a step-wise approach and an interdisciplinary agreement has been described before [29, 30]. Olthof et al. have demonstrated that interdisciplinary workflow optimisation including clarification of imaging request forms, subspecialisation of radiologists and structured reporting improves the quality of radiology reports in oncological patients [31]. Gormly reported on experiences with an oncological reporting concept that includes reporting templates with a layout comparable to SOR [32]. However, scepticism against structured reporting is prevalent among radiologists [33]. Raised concerns include the fear of interference with the natural process of image interpretation, non-feasibility for complex cases and cumbersome utilisation [33]. Our data on implementation of SOR in clinical practice show, however, that compliance with such structured reporting concepts and structured reporting tools aside from the RIS can be high even in settings with high reporting workload.

Web browser-based reporting tools can be used to generate structured reports. Several authors have used a commercial online reporting solution that is primarily designed to generate structured reports containing semantic sentences using predefined text phrases from itemised point-and-click data entry [5, 9, 28, 34–36]. Pinto dos Santos et al. have developed an open-source reporting platform that is compliant with IHE Management of Radiology Report Templates profile and stores report information in an additional database aside from RIS to facilitate data analysis [37].

We used a different software application programmed in-house in order to create a reporting template for oncological follow-up imaging that fits best to local clinical and radiological demands and represents the locally approved reporting concept in detail. SOR are compiled within the software application and are exported to the RIS as plain text via copy and paste. SOR are stored in RIS only and not in an additional SOR database. Scientific analyses of information contained in SOR can be performed using language processing techniques after extracting reports from RIS. In our opinion, scientific analyses of a separate SOR database would require data consistency with RIS, in which the report finally has to be cleared. Data consistency, however, is difficult to establish considering (1) an obligatory RIS-based multistep reporting process including primary reporting by an assistant radiologists and approval by a board-approved radiologist and (2) necessity of a technological means that SOR database and RIS database reports are identical. On the other hand, lack of a separate SOR database impedes inclusion of data from previous reports into the current report to ease reporting, e.g. inclusion of reference lesion measurements in long-term follow-up.

### Limitations

The implementation of our reporting concept has constraints. The influence of SOR implementation on reporting times was not determined. The experiences of radiologists in generating the reports and objective parameters of report turnaround times were not investigated and should be investigated prospectively. For quantitative assessment of long-term follow-up, tumour measurements recorded in reports of previous examinations have to be considered, because in SOR only the last prior tumour measurements are included. The objective impact of SOR on clinical decision-making was not assessed. In the analysis of satisfaction with reports, only reports from patients with metastatic colorectal cancer have been investigated as a pars pro toto. The number of reports that has been analysed for referring physicians’ satisfaction was small.

### Conclusion

Department-wide structured reporting for general follow-up imaging studies performed in metastatic cancer patients for assessment of anticancer treatment efficacy is feasible using a dedicated IT reporting tool. Satisfaction of referring oncologist with report quality is superior for structured reports compared with conventional reports.

## Data Availability

The datasets used and analysed during the current study are available from the corresponding author on reasonable request.

## Abbreviations

CR: Conventional report
CT: Computed tomography
HTML: HyperText Markup Language
IT: Information technology
mCRC: Metastatic colorectal cancer
MRI: Magnetic resonance imaging
RECIST: Response Criteria In Solid Tumours
RIS: Radiology information system
SOR: Structured oncology report
